# Improved Coefficient Recovery and Its Application for Rewritable Data Embedding

**DOI:** 10.3390/jimaging7110244

**Published:** 2021-11-18

**Authors:** Alan Sii, Simying Ong, KokSheik Wong

**Affiliations:** 1 School of Information Technology, Monash University Malaysia, Subang Jaya 47500, Malaysia; alan.sii@monash.edu; 2 Faculty of Computer Science and Information Technology, Universiti Malaya, Kuala Lumpur 50603, Malaysia; simying.ong@um.edu.my

**Keywords:** coefficient recovery, segmentation, adaptive, rewritable, DCT

## Abstract

JPEG is the most commonly utilized image coding standard for storage and transmission purposes. It achieves a good rate–distortion trade-off, and it has been adopted by many, if not all, handheld devices. However, often information loss occurs due to transmission error or damage to the storage device. To address this problem, various coefficient recovery methods have been proposed in the past, including a divide-and-conquer approach to speed up the recovery process. However, the segmentation technique considered in the existing method operates with the assumption of a bi-modal distribution for the pixel values, but most images do not satisfy this condition. Therefore, in this work, an adaptive method was employed to perform more accurate segmentation, so that the real potential of the previous coefficient recovery methods can be unleashed. In addition, an improved rewritable adaptive data embedding method is also proposed that exploits the recoverability of coefficients. Discrete cosine transformation (DCT) patches and blocks for data hiding are judiciously selected based on the predetermined precision to control the embedding capacity and image distortion. Our results suggest that the adaptive coefficient recovery method is able to improve on the conventional method up to 27% in terms of CPU time, and it also achieved better image quality with most considered images. Furthermore, the proposed rewritable data embedding method is able to embed 20,146 bits into an image of dimensions 512×512.

## 1. Introduction

Despite the emerging technologies, such as 3D video and augmented reality, the still image remains one of the most popular forms of media in use. Most, if not all, smart devices are equipped with a camera, hence making images easily accessible to everyone. In addition to the simple capturing process, digital images can also be edited and transmitted by using any network-enabled smart device. For storing and representing an image, some chose to store the continuous sensed data (produced by the light sensitive sensors in a camera) in the raw format to preserve all detail that can be captured by the sensor. However, in most cases, the image data are encapsulated by using some image formats for storage and transmission purposes. Some of the popular formats include JPEG (Joint Photographic Expert Group), GIF (Graphics Interchange Format), PNG (Portable Network Graphics), and TIFF (Tag Image File Format).

JPEG is the arguably the most commonly utilized image file standard due to its good rate–distortion trade-off. It is also compatible with most browsers, image viewers, and handheld devices. Therefore, research revolving around the JPEG standard has been receiving much attention since its inception in 1995 [1]. Discrete cosine transformation (DCT), which converts a block of pixel values into a weighted linear combination of basis patterns, is one of the main processes in JPEG. DCT produces two types of coefficient (weight), the DC and AC coefficients. In order to further reduce redundancy, quantization is applied to these coefficients, and the quantized coefficients are re-arranged into a more compact representation using the zig-zag scanning method then encoded by using zero run length coding. The output is eventually coded by using entropy coding, i.e., Huffman coding, to produce the JPEG compliant image.

These encoded JPEG images are then stored or transmitted for their intended usage. However, from time to time, the JPEG images might have encountered information loss [2] due to transmission error or information loss in the storage device (e.g., due to a bad sector in a hard disk [3]). Among the information available in a JPEG file, the DC coefficients are particularly important because they carry the overall intensity of the DCT blocks of the image. Therefore, in 2006, Uehara et al. [4] proposed a coefficient recovery method to recover the DC coefficients using their identified compressed image properties. Although Uehara et al.’s method successfully recovers the DC coefficients under various JPEG compression factors, it is suffering from the overflow and underflow problems [5].

Later, Li et al. [6] improved Uehara et al.’s method by using linear optimization approach and further extended its application to recover both DC and AC coefficients. This method can recover the overall structure and intensities when DC and AC coefficients are missing, and the recovered output images attain high SSIM values. Using subjective evaluations, Li et al.’s method often produced output images with narrow ranges of contrast. However, the computational complexity of the proposed linear optimization approach is high because a large number of equations and inequalities are taken into consideration during the recovery process.

Therefore, Ong et al. [7] proposed to divide the full-size image optimization problem into multiple scaled down problems. Specifically, from the image of interest (where some of its coefficients are missing), a sketch [8] of the image is produced. Otsu’s segmentation is then applied on the sketch to divide the image into smaller segments, where the pixels with similar intensities are grouped. Each segment is then an independent problem, and the missing coefficients in a segment are recovered by using the linear optimization approach put forward by Li et al. [6]. This segmentation process not only helps to reduce the number of constraints, but also restricts the solution space to a smaller region. Hence, the processing duration for Ong et al.’s method is 3–4 times faster than [6].

Interestingly, the possible applications of all the aforementioned works [4,5,7] are not limited to coefficient recovery only. These recovery/prediction works can be further extended to the cryptography/steganography algorithm attack [4,9], data embedding in encrypted images [10,11], forgery detection [12], and others. Among them, data embedding can be deployed to manage images, for example, hyperlinking related media to the image, fingerprinting, or enabling extra features for premium users, to name a few options. Specifically, data embedding is a process that modifies the carrier medium (e.g., image), aiming to encode as much data as possible while maintaining the fidelity of the original carrier. Depending on the application, the embedded data serves different purposes.

The conventional data embedding methods aim to maintain the quality of the carrier image. Most methods achieve their goals by making small changes to the image. However, when data can be recovered with a certain level of accuracy, why not just replace the recoverable data with the data to be embedded? In addition, if an image of higher quality is required, the embedded data can be scrapped, and data removed earlier (to make room for data embedding) can be recovered. Therefore, in this work, we will first revisit Ong et al.’s method [7] and Tan et al.’s method [10] to solve two problems. Although Ong et al.’s method improves Li et al.’s [6] method in terms of CPU time, the true capability of divide-and-conquer has not been harvested due to the low performance in Otsu’s method (adopted in Ong et al.’s method) in image segmentation. On the other hand, although Tan et al.’s method is designed to embed data into an encrypted JPEG image, the quality of the decrypted-recovered image is low, because it simply removes all the relevant coefficient(s) in every region for data embedding without considering the distortion caused.

The rest of this paper is structured as follows: Section 2 reviews the coefficient recovery methods proposed by Li et al. and Ong et al., followed by the rewritable data embedding method by Tan et al. The proposed improvement and rewritable data embedding methods are detailed in Section 3. Experiment results are then presented in Section 4, and Section 5 concludes this article.

## 2. Related Work

In the JPEG image encoding process, an input image will first be divided into 8×8 non-overlapping pixels blocks. These blocks are known as Minimum Coded Units (MCUs). For each MCU, DCT is applied to produce 8×8 coefficient blocks, where the top-left coefficient is the DC coefficient, and the rest are the AC coefficients. The DC coefficient carries the overall intensity of the MCU, and AC coefficients are used to store the weights of the 63 DCT basis vectors (i.e., block patterns).

To the best of our knowledge, the earliest work on coefficient recovery was proposed by Uehara et al. [4]. In particular, Uehara et al.’s method utilizes the remaining AC coefficients to recover the missing DC coefficient because the range of the DC coefficient in a block is constrained by the pixel values generated by the AC coefficients (viz., the mean-removed pixels). In addition, to ensure the global feature of the image, Uehara et al.’s method also considers the close relationship between vertical and horizontal pixels while recovering the DC coefficients. In their work, Uehara et al. successfully performed an attack on DC-encrypted images by revealing (recovering) the DC coefficients.

Later, Li et al. extended Uehara et al.’s method in new directions, i.e., recovering both the DC and AC coefficients, by using linear optimization. Li et al. treated the missing coefficients problem as a minimization problem:

$$\begin{array}{cc} \mathrm{minimize}\; & \;\sum h_{x,y,x^{\prime },y^{\prime }}\\ \mathrm{subject}\;\mathrm{to}\; & \;I\left(x,y\right)-I\left(x^{\prime },y^{\prime }\right)\leq h_{x,y,x^{\prime },y^{\prime }},\\ \; & \;I\left(x^{\prime },y^{\prime }\right)-I\left(x,y\right)\leq h_{x,y,x^{\prime },y^{\prime }},\\ \; & \;I=A.J,\\ \; & \;I_{\min}\leq I\left(x,y\right)\leq I_{\max},\\ \; & \;J(u,v)=J*(u,v), \end{array}$$

where I(x,y) denotes the pixel value at (x,y), $I(x^{\prime },y^{\prime })$ is the neighboring pixel value of I(x,y), $h_{x,y,x^{\prime },y^{\prime }}$ is the difference for a pair of neighboring pixels, *A* is the DCT transformation matrix, J(u,v) is DCT coefficient value at (u,v), and J∗(u,v) is the known DCT coefficient value. The generalization using linear optimization in [5] is more flexible and convenient, as it can recover more coefficients and reduces the implementation complexity.

However, using Li et al.’s approach to solve a full-image recovery of coefficients problem produces many constraints, and the solution space is wide. In other words, it incurs high computational complexity. Therefore, Ong et al. [7] proposed to divide the full-image problem into multiple smaller and independent optimization problems to reduce the computational cost. An intuitive segmentation technique, i.e., Otsu’s method, was utilized in [7] to divide an image into segments. In each segment, the same objective function was utilized but with a smaller number of constraints. Within the segment, it was also found that the solution space for the linear optimization algorithm is relatively smaller than that of [5] because the pixels values are very similar. Based on the results reported in [7], this divide-and-conquer approach is able to reduce the computational cost by 3∼4 times compared to that of [5].

Besides using the recovery approach to break the encryption technique, as demonstrated in [4], this approach has been utilized to embed data. Tan et al.’s method [10] removes chosen group of coefficients and shifts the remaining coefficients to the upper part of the MCU, to vacate the space in storing external data. This shifting process also contributes to their goal of degrading the image quality, which eventually leads to perceptual encryption. The external data are then represented by using the Huffman codewords, and these codewords corresponding to the external data are placed in the vacated spaces in the MCUs. On the receiver side, the inserted external data are extracted, and the shifted coefficients are put back to the same positions, along with other decryption operations. Finally, the removed coefficients can be approximated using the recovery algorithm as in [7].

## 3. Proposed method

In this work, we improve Ong et al.’s image coefficient recovery method [7], and then propose a rewritable data hiding method which exploits the fact that the removed coefficients can be recovered with high accuracy. The details are presented in the following subsections.

### 3.1. Improved Coefficient Recovery

As reported by Ong et al. in [7], an improvement of 3∼4 times over Li et al.’s method [6] in terms of CPU time has been attained. The main ingredient behind Ong et al.’s method is the divide-and-conquer strategy. Specifically, the image of interest is divided into non-overlapping patches, and the missing coefficients within a patch are recovered independently from other patches.

In Ong et al.’s method, the patches are defined based on the remaining coefficients in the image. Specifically, the energy induced by the remaining coefficient in each 8×8 block plays the role of a pixel [8], and the resulting matrix (which is 1/8×1/8 of the original size in each dimension) of values is linearly scaled to put them in the range of [0,1] to form the energy image *E*. Otsu’s method is then applied to divide the energy image into the background and foreground regions.

**Figure 1:**
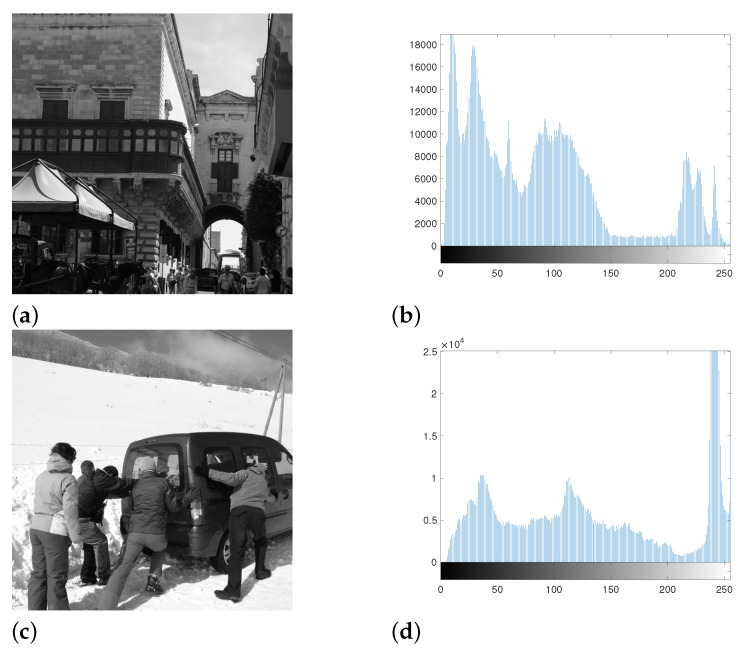
Natural images ((**a**) image N11 and (**c**) image N18)) and their distributions of pixel values ((**b**) for image N11 and (**d**) for image N18). Both histograms have more than 2 peaks.

However, there is an issue, because Otsu’s method operates based on the assumption that the pixel distribution has two peaks (i.e., bimodal). In fact, most natural images do not exhibit that distribution. For example, see Figure 1 for the distributions of pixel values of two test images from the BOSSbase dataset [13]. Therefore, depending on the statistical features of the image of interest, Otsu’s method may not be suitable, and hence the background/foreground separation output is expected to be suboptimal. As a result, the true potential of the divide-and-conquer approach cannot be maximally harvested this way.

To address this issue, we adopt the adaptive segmentation method by Bradley and Roth (referred to as BR’s method) [14]. This particular adaptive segmentation method is adopted because it is lightweight; hence, it will not add significant complexity to the coefficient recovery process. The main idea in BR’s method is to compare the pixel of interest I(x,y) against the average pixel intensity value of a s×s neighborhood surrounding I(x,y). The computation is efficiently performed by adopting the concept of integral image *Q*, where each value in the integral image $Q(x_{1},y_{1})$ refers to the summation of the pixel values from the top-left pixel to the $\left(x_{1},y_{1}\right)$-th position. In other words,

(1) $$Q\left(x_{1},y_{1}\right)=\sum_{x=1}^{x_{1}}\sum_{y=1}^{y_{1}}I\left(x,y\right).$$

Subsequently, the average value, denoted by $\hat{I}\left(x,y\right)$, is compared with I(x,y) to determine whether I(x,y) belongs to the background (black) or foreground (white) region of the image. Specifically,

(2) $$I\left(x,y\right)\in \left\{\begin{array}{cc} \; & F\;\mathrm{if}\;I\left(x,y\right)\geq p\times \hat{I}\left(x,y\right);\\ \; & B\;\mathrm{otherwise}, \end{array}\right.$$

where *B* and *F* denote the background and foreground (regions) sets, respectively, and *p* denotes a scaling factor ranging from 0 to 1. Once the patches are defined, Li et al.’s method [6] is applied to each patch to recover the coefficients.

**Figure 2:**
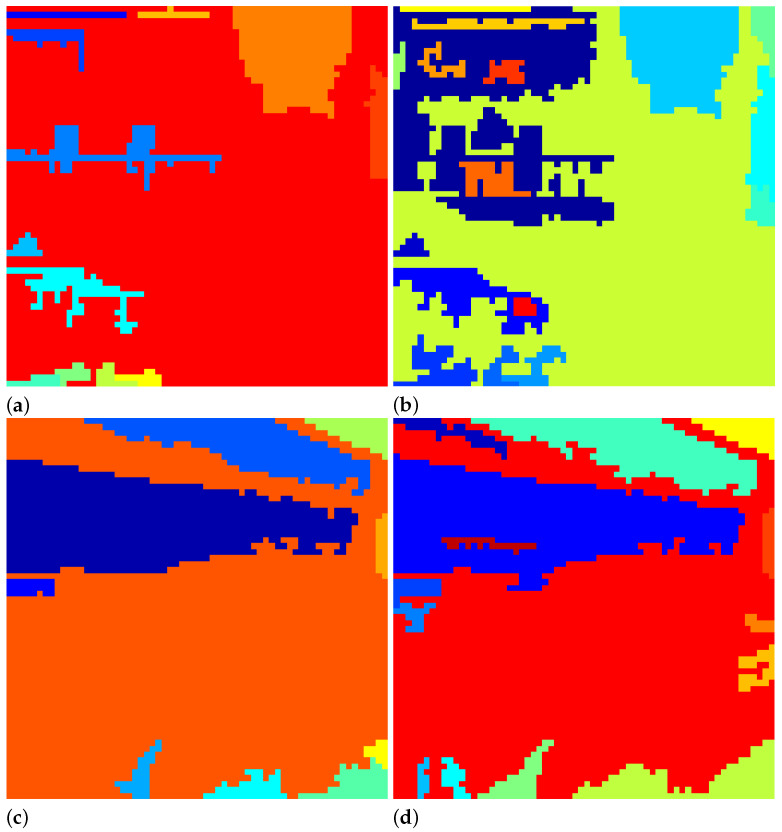
Segments produced by using Otsu’s method (**a**,**c**) and BR’s method [14] (**b**,**d**). The first row shows the results for image N11, and the second row shows the results for image N18. The segments are colored for illustration purposes. Refer to Figure 1 for the original images.

The segmentation results using Otsu’s method and BR’s method are shown in Figure 2. It is apparent that BR’s method produces: (a) a higher number of segments (or patches) and (b) better segment boundaries. In particular, (a) allows more parallelism since the patches are handled independently (hence shorter CPU time), and (b) preserves the quality of the image at the patch level.

### 3.2. Rewritable Data Embedding

In this section, we put forward a rewritable data embedding method by exploiting the fact that coefficients could be removed from an image *I* and later recovered to form the image $I^{r}$, where *I* and $I^{r}$ are visually similar. Here, the coefficients could be obtained from an image *I* which is stored in the form of a matrix of pixel values, or obtained by partially decoding a JPEG bit stream. Our method is *rewritable* because the loss of information only occurs in the first round of embedding, i.e., when the selected coefficients in *I* are modified to accommodate the data (message, μ). The embedded data can be removed and the coefficients could be recovered. When the image with the recovered coefficients, i.e., $I^{r}$, is used in turn as the host image for data embedding, one can remove the embedded data and recover the coefficients to perfectly reproduce $I^{r}$, where no further information is lost.

To embed data, we first classify each 8×8 block based on how well the removed coefficients could be recovered. Specifically, we form the energy image *E* by computing:

(3) $$E\left(i,j\right)=\sum_{a\neq 1,2,3}AC_{a}\left(i,j\right),$$

where $AC_{a}\left(i,j\right)$ refers to the *a*-th AC coefficient (in zigzag order) in the (i,j)-th 8×8 block. Subsequently, *E* is scaled by dividing T=max{E(i,j)}, i.e., E←E/T, so that the resulting values in *E* ranges from 0 to 1. BR’s method is then executed on *E* to label each pixel value, i.e., “0” for background and “1” for foreground. A set of connected pixels (with respect to 8-neighborhood) forms a patch $P_{d}$, where d=1,2,⋯. Therefore, there can be multiple patches of background and foreground (i.e., disconnected regions).

Specifically, $P_{d}\left(i,j\right)=1$ implies that the (i,j)-th 8×8 block belongs to the *d*-th patch, while $P_{d}\left(i,j\right)=0$ implies otherwise. By construct, most values in each patch $P_{d}$ will be “0”. In addition, we do not distinguish background from foreground patches. However, as long as their removed coefficients can be recovered within a predetermined precision (denoted by τ), they can be selected for data embedding purposes. The proposed coefficient recovery method is then executed to recover the removed coefficients.

To quantify the precision, the difference between the original coefficient (denoted by $AC_{a}$) and recovered coefficient (denoted by $AC_{a}^{r}$) is computed; i.e.,

(4) $$\delta_{a}\left(i,j\right)=AC_{a}\left(i,j\right)-AC_{a}^{r}\left(i,j\right).$$

Subsequently, the mean square error (MSE) for the *d*-th patch is computed as:

(5) $$MSE\left(P_{d}\right)=\frac{1}{|P_{d}|}\sum_{a=1}^{3}\sum_{i}\sum_{j}\delta_{a}^{2}\left(i,j\right)⊗P_{d}\left(i,j\right),$$

where ⊗ denotes the element-wise multiplication, and |X| denotes the cardinality of the set *X*. The patches with $MSE(P_{d})<\tau$ are labeled as *usable*; otherwise, *unusable*. Subsequently, the patches $P_{d}$ are sorted based on size (i.e., $|P_{d}|$) in decreasing order. Usable patches within the 20 largest patches are considered for data embedding, and the remaining patches are left unmodified. Here, a 20-bit array (denoted by *M*) is constructed to record which patch is usable (i.e., “1”) or unusable (i.e., “0”). For example, M(4)=0 implies that all 8×8 blocks belonging to the 4-th largest patch are usable for data embedding. Subsequently, *M* is communicated to the receiver, either through some reserved space in the image or through a separate communication channel.

**Figure 3:**
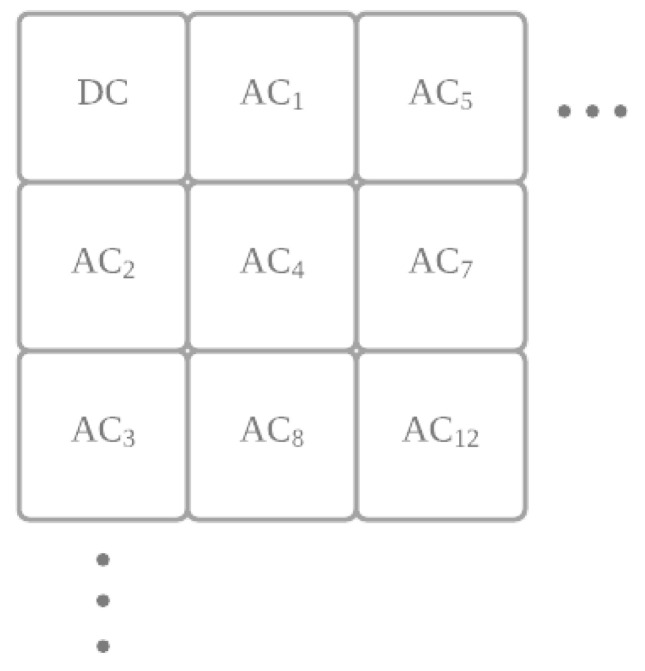
The naming convention adopted in this work for DC and AC coefficients. $AC_{1}$, $AC_{2}$, and $AC_{3}$ are adopted for data embedding.

Next, $AC_{1}$, $AC_{2}$, and $AC_{3}$ from each of the 8×8 blocks (see Figure 3) in the 20 largest usable patches are utilized for data embedding purposes. We divide the message μ into *l*-bit segments, each denoted by $\mu_{k}$, k=1,2,⋯,⌊|μ|/l⌋. To embed data, the *l* least significant bits of $AC_{1}$ are replaced by $\mu_{k}$, and the process is repeated for $AC_{2}$ and $AC_{3}$ using the following message segments, i.e., $\mu_{k+1}$ and $\mu_{k+2}$. In essence, each 8×8 block in a usable patch will hold 3×l bits from the message μ. The process is repeated for all blocks until all message segments $\mu_{k}$ are processed, or all usable blocks are exhausted. Note that more features could be added to the embedding process, for example, randomizing the sequence in which we process the message segments, i.e., from $\mu_{k}$ to $\mu_{k^{\prime }}$, or encrypting them to $\mu_{k}^{\prime }$ before embedding, or both. In addition, some 8×8 blocks in the usable patch could also be skipped—i.e., not utilizing all blocks for data embedding.

In any case, the modified coefficients (now containing data) in the usable patches are combined with the unmodified coefficients to form the processed image $I^{+}$.

### 3.3. Data Extraction and Image Recovery

First, $AC_{1}$, $AC_{2}$, and $AC_{3}$ are removed from the received image $I^{+}$ and the energy image *E* is formed. Next, the background and foreground patches $P_{d}$ are formed by applying BR’s method [14] on *E*. Subsequently, the patches are sorted based on size in decreasing order, and *M* is consulted to determine whether a patch is usable or unusable. For each 8×8 block in a usable patch, the *l* least significant bits of $AC_{1}$, $AC_{2}$, and $AC_{3}$, will produce three message segments. In other words, the *k*-th 8×8 blocks will produce $\mu_{3k},\mu_{3k+1}$, and $\mu_{3k+2}$. The process is repeated for all blocks in each usable patch. Subsequently, the message μ is reconstructed by concatenation:

(6) $$\mu =\mu_{1}\left|\right|\mu_{2}\left|\right|\mu_{3}\left|\right|\cdots .$$

Finally, to produce an approximation of the original image $I^{r}$, Li et al.’s coefficient recovery method [6] is invoked to recover $AC_{1}$, $AC_{2}$, and $AC_{3}$ for each usable patch. When we put the recovered coefficients (i.e., $AC_{1}^{r}$, $AC_{2}^{r}$, and $AC_{3}^{r}$) together with the unmodified coefficients (i.e., those from the unusable patches and the unmodified coefficients in the usable patches), we obtain an approximation of the original image.

## 4. Experiment

The proposed improvement on Ong et al.’s recovery method and the proposed rewritable data embedding method were implemented on MATLAB (Version 2020b) running on an AMD Ryzen 7 4800H PC with 8 GB of memory (Windows 10). Experiments were conducted by using the same 20 test images (512×512) from BOSSbase dataset [13], which are available online [15]. For all experiments, the first three AC coefficients, i.e., $AC_{1}$, $AC_{2}$, and $AC_{3}$ were removed, utilized to embed data, and eventually recovered. The data embedded in the experiments were randomly generated by using the pseudo-random binary generator (PRBG) using a fixed seed. For BR’s method [14], the threshold p=0.8 is set and for the proposed rewritable data embedding method, the threshold τ is set based on a ratio, i.e., 20% of the mean square error (MSE) values for all recovered coefficients. For data embedding, among the 20 largest patches, those with MSE value above τ are skipped, i.e., neither removal nor data embedding was carried out in those patches. After data extraction, all the embedded binary bits were compared with the binary sequence generated by using the same seed as in data embedding, and it was confirmed that the recovered data contained no errors.

### 4.1. Coefficient Recovery

First, we examined the quality of resulting images after the missing coefficients were recovered by using the proposed improvement method (denoted by *Adaptive* Method) and Ong et al.’s method [7]. In terms of the segmentation process, BR’s method was able to produce clearer and more refined boundaries for the images considered. For example, for the energy image of N11 shown in Figure 2, the contour of the balcony was captured more accurately when using BR’s method, whereas Otsu’s method grouped several distinctive regions into one patch. To quantify the quality of the recovered images, the PSNR (dB) and SSIM [16] scores were recorded in Table 1. The results suggest that the proposed method and Ong et al.’s method achieved similar image quality, although the proposed method showed marginally better image quality—18 out of 20 for PSNR and 16 out of 20 for SSIM. The average PSNR/SSIM for the proposed method and Ong et al.’s method [7] were 30.15 dB/0.9248 and 28.99 dB/0.9207, respectively.

**Figure 4:**
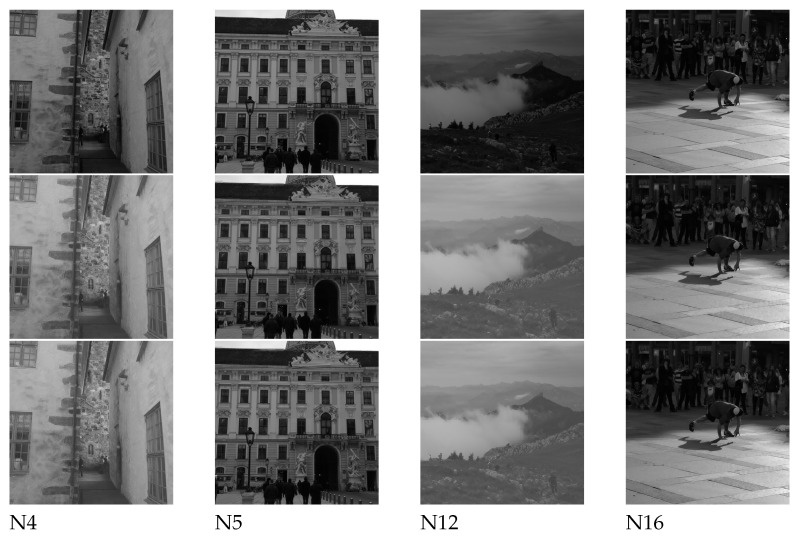
Original images (**first row**), recovered images produced by using Ong et al.’s method [7] (**second row**) and the proposed method (**third row**).

To further confirm this observation, selected images are shown in Figure 4. By visual inspection, Ong et al.’s method and the proposed method produced images which are visually similar to the original image, hence confirming their ability to recover the removed coefficients. It is noticed that for both recovery methods, the recovered images tend to be brighter (see N4 and N12). Upon further investigation, it was observed that images N4 and N12 are both simple in texture, having less visual details in most parts of the image (i.e., the wall for N4 and the cloud for N12). In such smooth blocks, there were lesser high frequency DCT coefficients, and most energy is concentrated on the low frequency DCT coefficients, representing the smoother pattern of the DCT block. However, the low frequency DCT coefficients (i.e., first 3 AC coefficients) are removed in our experiments. Hence, the linear solver might face difficulties in finding the optimum solution and predict the intensity for such DCT blocks, because only a very limited number of coefficients are available as the reference. Therefore, it can be observed that the recoverability of the coefficients in these kinds of images in both methods are not as accurate as the other textured images.

Second, we considered the number of patches produced by BR’s method and Otsu’ method. The results are recorded in Table 1. As expected, the number of patches produced by BR’s method was consistently higher than that of Otsu’s method, except for image N13. In particular, the largest difference was an increment of 17 patches as observed in image N6. On average, BR’s method produced 17 patches, whereas Otsu’s method in Ong et al.’s proposal produced 9.6 patches.

Third, the CPU times were compared, the results of which are also recorded in Table 1. The CPU time here indicates the computational time used to solve the linear programming problems of all patches in the image. For all 20 images considered, the proposed improvement always completed the coefficient recovery task in a shorter period of time (see the bold values in Table 1). The percentage of improvement ranged between 1.35% and 27%, and the average improvement was 10.02%. In general, a significant improvement was observed for images, with large differences in terms of the number of patches. Even Ong et al.’s method produced more patches than the proposed adaptive method (see N13), and when both methods had similar number of patches (i.e., ±2 to 3 patches, see N1 and N20), the proposed adaptive method still required less CPU time—viz., solving the linear programming problem in a shorter time span. This also shows the effectiveness of using an adaptive segmentation method in the linear programming solver as compared to the conventional segmentation method, which uses a global threshold.

**Figure 5:**
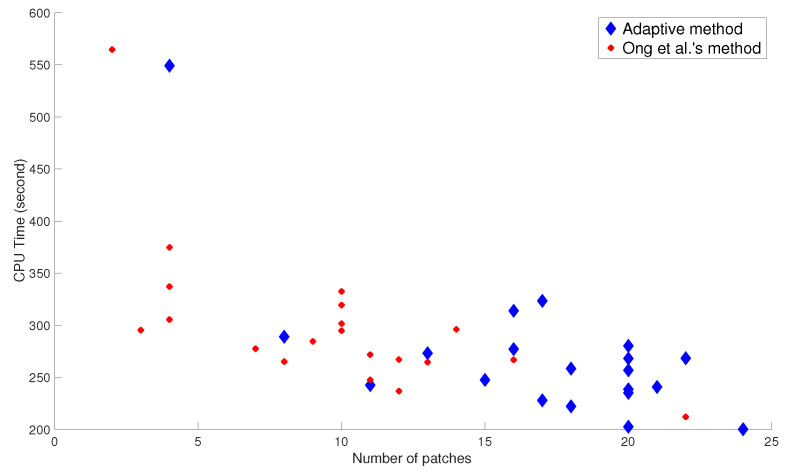
The graph of CPU time against number of patches.

For completion of discussion, the graph of CPU vs. number of patches is shown in Figure 5. It is shown that the CPU time is negatively correlated with the number of patches; i.e., CPU time decreased as the number of patches increased. Therefore, having larger number of patches implies, in general, less CPU time.

### 4.2. Rewritable Data Embedding

In this subsection, we evaluate the performance of the proposed rewritable data embedding method. First, for results of the embedding capacity, the number of usable 8×8 blocks, and the number of patches are recorded in Table 2. The 20 largest patches in each image were considered for data embedding. However, not all of the patches are qualified (viz., not satisfying the condition $|P_{d}|>\tau$). As recorded in Table 2, the number of qualified patches ranged from 4 to 20; and on average, 17 of them were usable. We observed that the number of usable patches does not imply higher embedding capacity. This is because the patch size (i.e., number of 8×8 blocks belonging to each patch) dictates the embedding capacity, and the patch size is varies depending on the texture of the test image. In particular, N1 only had four qualified patches, but the number of qualified blocks was >2000. For N13, although all 20 largest patches were usable, the number of qualified blocks was only 1704. Note that N1 produced larger patches due to its smoother texture and fewer edges, whereas N13 produces many smaller patches because it has more complex texture and more edges.

This also explains the reason behind the variations in embedding capacity for images where all largest 20 patches are usable, e.g., see N6 (31,779 bits) and N13 (15,336 bits). Based on our observations, N6 achieved the highest embedding capacity because it has fewer edges (viz., larger patches), and its slightly rough texture (that can pass the precision test) is suitable for data embedding purposes. If an image has less texture (i.e., smooth), the distortion caused by data embedding will be obvious, hence making most of the patches in smooth images such as N1 fail in the precision test. Therefore, depending on the edges and textures of the test images, the embedding capacity of these test images ranged from 12,636 to 31,779 bits using the same threshold settings. On average, 20,146 bits could be embedded into each image. In other words, 2238 8×8 blocks were usable.

Second, let $I^{-}$ denote the image with its coefficients *AC*${}_{1}$, *AC*${}_{2}$, and *AC*${}_{3}$ removed. Similarly, let $I^{+}$ denote the image after embedding data into $I^{-}$ using the proposed method. However, for the non-usable patches in *I*, the coefficients *AC*${}_{1}$, *AC*${}_{2}$, and *AC*${}_{3}$ were copied back into $I^{+}$. The quality of both $I^{-}$ and $I^{+}$ is also recorded in Table 2. For all images except four (i.e., N1, N4, N8, and N12), the image quality for $I^{+}$ is higher than that of $I^{-}$ (see the bold values in Table 2). Images N1,N4,N8, and N12 are the exceptions because they are smooth, as opposed to the other images, which contain objects with complex backgrounds and textures. The test images used in these experiments are shown in Appendix A. Furthermore, the image quality of $I^{+}$ is also affected by the total number of qualified blocks for data embedding and the embedded data. When there are less qualified blocks for data embedding, it implies that more AC coefficients remain intact, since there are more unqualified blocks. Moreover, three first AC coefficients are vacated to carry data; hence, the inserted data also affects the image quality. For the same reason, we observed that for some images, the quality reported here is higher than that of their counterparts, as reported in Table 1. In fact, the results reported in Table 1 serve to estimate the quality of the recovered image when all patches (blocks) are utilized for data embedding purposes.

For comparison purposes, Guo et al.’s method [17] was considered, due to the similarities of their work and ours. Although different image datasets were considered, Guo’s method was able to embed 2205/(256×256)=0.03365 bits per pixel, whereas the proposed method could embed 0.07685 bits pixel. In terms of image quality, on average, Guo’s et al.’s method [17] achieved 39.19 dB; the proposed method achieved 30.01 dB. Based on these outcomes, we conclude that our proposed method trades image quality for higher embedding capacity. In fact, higher image quality can be attained with the proposed data embedding method by: (a) setting a small threshold τ for MSE, or (b) using fewer AC coefficients for data embedding purposes.

**Figure 6:**
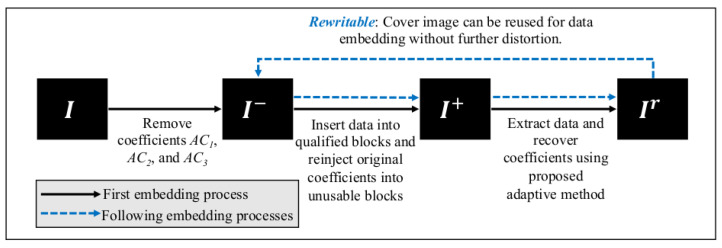
The rewritable process in the proposed method.

It is noteworthy that the proposed data embedding method is irreversible but rewritable. The rewriting process is illustrated in Figure 6. For a reversible data embedding method, the images *I* and $I^{r}$ (viz., image after data extraction and recovery) are always identical. However, in our proposed data embedding method using the adaptive coefficient recovery technique, information loss occurs when we generate $I^{-}$ from *I*. When the embedded data are removed from $I^{+}$, the proposed improved coefficient recovery method can be invoked to recover the coefficients removed earlier to form the recovered image $I^{r}$. When $I^{r}$ is in turn used for data embedding by using the same threshold value, the receiver will be able to form $I^{-}$, and subsequently reconstruct $I^{r}$. Therefore, the embedding-recovery cycle can be repeated without incurring further quality degradation as long as the same threshold (viz., τ) is in use. This is because the same coefficient recovery method is used in the process with reference to the unused coefficients, which stay intact all the time.

## 5. Conclusions

In this study, we first identified the problem caused by the use of Otsu’s segmentation method in Ong et al.’s coefficient recovery method [7]. Specifically, the bi-modal distribution assumption in Otsu’s method leads to suboptimal performance, where the true potential of divide-and-conquer cannot be fully harvested. Accordingly, we proposed to replace Otsu’s method by using an adaptive method put forward by Bradley and Roth [14]. In addition, we put forward a rewritable data embedding method by exploiting the fact that coefficients can be recovered when the neighboring coefficients remain intact. Among the largest 20 patches induced by Bradley and Roth’s segmentation method, the usable ones are exploited for data embedding purposes. The three least significant bits of each of the $AC_{1}$, $AC_{2}$, and $AC_{3}$ coefficients are replaced by the message bits to be embedded. Experiment results suggest that the proposed improved coefficient recovery method is able to achieve up to 27% improvement in terms of CPU time over Ong’s et al.’s method without compromising on image quality. In addition, the proposed method is able to embed, on average, 20,146 bits into each image. The proposed data embedding method is also rewritable.

As future work, we want to find a more secure and efficient way to encode the 20-bit location map or to eliminate the need for that map completely. We also aim to apply the proposed coefficient recovery method in the video domain.

## Figures and Tables

**Table 1:** Performance comparison between the proposed adaptive coefficient recovery method and Ong et al.’s method [7].

Image	Adaptive Method				Ong et al.’s Method				CPU Time
	PSNR	SSIM	No. of	CPU	PSNR	SSIM	No. of	CPU	Improvement
	(dB)		Patches	Time (s)	(dB)		Patches	Time (s)	(%)
N1	**25.59**	**0.9433**	**4**	**549.13**	25.49	0.9366	2	564.66	2.75
N2	**31.88**	**0.9497**	**16**	**277.17**	31.00	0.9410	10	301.63	8.11
N3	**31.66**	**0.9445**	**18**	**258.42**	31.64	0.9442	13	264.72	2.38
N4	**16.50**	**0.8881**	**18**	**222.23**	16.41	0.8873	12	236.81	6.16
N5	**30.29**	**0.9277**	**17**	**323.44**	30.00	0.9273	10	332.45	2.71
N6	37.06	0.9602	**21**	**240.80**	**37.12**	**0.9604**	4	305.56	21.19
N7	**33.50**	0.9230	**20**	**238.56**	33.11	**0.9372**	9	284.53	16.16
N8	**26.50**	**0.9624**	**24**	**200.23**	25.96	0.9601	7	277.55	27.86
N9	**33.33**	**0.9658**	**8**	**289.05**	33.14	0.9514	4	374.75	22.87
N10	**35.24**	**0.9600**	**17**	**227.97**	35.03	0.9592	11	247.58	7.92
N11	**31.87**	**0.9419**	**20**	**235.14**	25.81	0.9101	16	266.94	11.91
N12	**12.00**	0.6400	**11**	**242.56**	11.79	**0.6511**	8	265.23	8.55
N13	**33.50**	**0.9312**	20	**202.63**	33.11	0.9300	**22**	212.13	4.48
N14	**27.08**	**0.9288**	**22**	**268.38**	26.88	0.9278	4	337.06	20.38
N15	**32.98**	**0.9351**	**20**	**256.88**	32.54	0.9344	10	294.77	12.85
N16	**36.23**	**0.9508**	**20**	**268.14**	25.81	0.9382	11	271.80	1.35
N17	29.58	0.9422	**20**	**280.23**	**30.02**	**0.9501**	14	296.08	5.35
N18	**36.01**	**0.9692**	**16**	**313.91**	35.17	0.9639	10	319.34	1.70
N19	**30.47**	**0.9097**	**13**	**273.19**	28.81	0.8921	3	295.27	7.48
N20	**31.80**	**0.9214**	**15**	**247.58**	30.94	0.9122	12	267.06	7.29
Average	30.15	0.9248	17.0	270.78	28.99	0.9207	9.6	286.01	10.02

**Table 2:** Image quality after removal of coefficients ($I^{-}$) and after data embedding ($I^{+}$). Results for image quality are presented in the format of PSNR (dB)/SSIM. Capacity is recorded in the unit of bits. The number of qualified blocks and the number of qualified patches are recorded in the last 2 columns.

Image	$I^{-}$	$I^{+}$	Capacity	Qualified Blocks	Qualified Patches
N1	**26.06/0.8673**	25.51/0.9431	18,036	2004	4
N2	24.10/0.8381	**31.85/0.9494**	21,708	2412	16
N3	26.13/0.8293	**31.61/0.9350**	26,325	2925	18
N4	**28.95/0.8504**	16.38/0.8571	18,063	2007	18
N5	24.32/0.7709	**30.27/0.9275**	24,516	2724	17
N6	28.58/0.8787	**37.05/0.9600**	31,779	3531	20
N7	28.48/0.7967	**33.49/0.9226**	21,519	2391	20
N8	**33.60/0.9196**	26.49/0.9619	23,355	2595	20
N9	22.61/0.8059	**33.28/0.9650**	12,636	1404	8
N10	27.47/0.8470	**35.24/0.9597**	15,255	1695	17
N11	23.54/0.7833	**31.84/0.9410**	16,173	1797	20
N12	**36.04/0.9344**	11.60/0.6340	17,118	1902	11
N13	29.91/0.7995	**33.15/0.9304**	15,336	1704	20
N14	24.77/0.8248	**27.06/0.9281**	22,977	2553	20
N15	25.96/0.8097	**32.93/0.9346**	22,491	2499	20
N16	28.61/0.8314	**36.20/0.9501**	23,328	2592	20
N17	23.30/0.8165	**29.55/0.9420**	20,871	2319	20
N18	23.30/0.8043	**35.30/0.9644**	17,145	1905	16
N19	24.67/0.7776	**30.39/0.9093**	16,254	1806	13
N20	25.66/0.7602	**31.04/0.9171**	18,036	2004	15

Note: Quality of the image after coefficient recovery can be found in Table 1.

**Figure A1:**
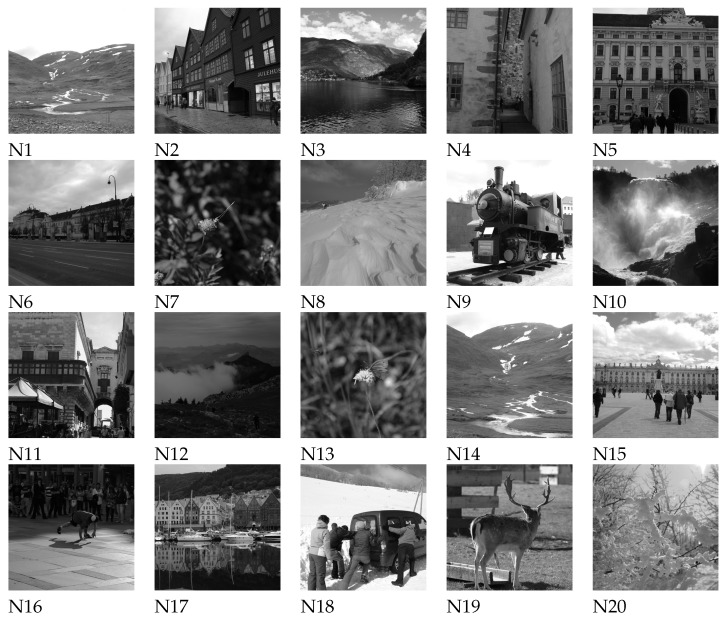
The test images (N1 to N20) utilized in the experiments.

## Data Availability

Not Applicable.

## Appendix A

The 20 test images utilized in all the experiments are provided in Figure A1. The images were obtained from the BOSSbase dataset [13].
